# Supplementary material for: Adaptive and freeze-tolerant heteronetwork organohydrogels with enhanced mechanical stability over a wide temperature range
Source: Nat Commun. 2017 Jun 22;8:15911. doi: 10.1038/ncomms15911 (PMC5489716; doi:10.1038/ncomms15911)
Supplement: Supplementary Information [file ncomms15911-s1.pdf]

Type of file: PDF
Size of file: 0 KB
Title of file for HTML: Supplementary Information
Description: Supplementary Figures, Supplementary Tables and Supplementary Methods

Type of file: PDF
Size of file: 0 KB
Title of file for HTML: Peer Review File
Description:

Supplementary Methods

Materials. Preparing poly(N,N-dimethylacrylamide) (PDMA) hydrogel need the following reagents: N,N-dimethylacrylamide (DMA) as monomers, N,N'-methylenebis (acrylamide) (BIS) as crosslinking agents, ammonium persulfate (APS) as initiators, and de-ionized water (DI-water) from Milli-Q Reference. Unless otherwise noted, all reagents were analytical reagents and used as received from J&K Scientific.

Organogel polymerization precursors consisted of n-butyl methacrylate (BMA), lauryl methacrylate (LMA) as monomers, ethylene glycol dimethacrylate (EGDMA) as crosslinking agents, 2,2-diethoxyacetophenone (DEAP) as photoinitiator, and anhydrous ethanol as solvent (Sinopharm Chemical Reagent Co. Ltd).

AFM measurement. A closed-loop scanner (5500 AFM, Keysight Technologies, Chandler, AZ, USA) was used to collect the topography data based on contact mode at room temperature. The spring constant of the cantilever (MSCT contact microlever, Bruker, Karlsruhe, Germany) is 0.03 N/m. The scanning area was set at 5×5 μm^2. The scanning rate was 0.8 Hz. The organohydrogel samples were fabricated based on H2 in Supplementary Table 1 for hydrogel and O1 in Supplementary Table 2 for organogel precursor, and the OPN/HPN ratio was at ~1.7. The *in-situ* scanning of organohydrogel in water was carried out to investigate the topography of water-equilibrated surface; and the sample surfaces were covered with the Milli-Q pure water cautiously. For oil equilibrated surfaces which were pretreated by n-dodecane overnight, and the scanning was performed in air at room temperature. The low volatility of n-dodecane was able to prevent surfaces from deforming. First-order fitting was applied to topography images to remove low-angle tilt of imaging surface. Then second-order polynomial background correction was performed to remove the surface distortion based on SPM analyzing software Gwyddion.

SEM characterization. The hydrogel and organohydrogel samples were swollen in distilled water to a saturation state. Then freeze them quickly in liquid nitrogen, followed by lyophilization at -85 °C. A field-emission scanning electron microscope (JSM-7500F, Japan) was used for characterizing the porous structures of the hydrogel and organohydrogel samples.

Preparation of organohydrogel coated mesh for smart separation. The organohydrogel-coated meshes were prepared by immersing the Cu mesh-200 into

hydrogel precursors for about 15 s to form a stable hydrogel layer coating on Cu mesh. The precursors of hydrogel consisted of DMA, BIS, APS, and DI-Water (Supplementary Table 1-H2). Then, the mesh was dehydrated by acetone repeatedly. The dehydrated hydrogel coated mesh was further swollen by the organogel precursor (Supplementary Table 2-O1), and placed under UV-irradiation for 30 min. The as-prepared organohydrogel coated mesh was rinsed repeatedly with acetone to remove residual impurities.

Method for adhesion measurement of Cu mesh. The adhesive force measurement of oil/water droplet on the mesh surface was conducted using a high sensitivity micro-electromechanical balance system (Data-Physics DCAT 11, Germany). Firstly, a liquid droplet (about 5 μL) was first loaded under a copper cap, which hung on the microbalance. The measured surfaces which placed on the balance table were controlled to approach the hung liquid droplet at the speed of 0.05 mm/s. Until the liquid droplet contacted the surface and deformed, the balance table moved down making the measured surface detach. The change of force was simultaneously recorded and the peak data of the receding curve was the adhesion force. A CCD camera was used to record images during the measurement process.

Method for smart separation of organohydrogel-coated meshes. The as-prepared mesh was fixed between two glass tubes. The diameter of the tube was 25 mm. The oil/water mixtures (vol/vol = 1/1) were poured onto the as-prepared mesh. The separation was achieved by gravity of the liquids. The oil concentration of the original oil/water mixtures and the collected water after separation were measured by the infrared spectrometer oil content analyzer (CY2000, China). Specialized tetrachloromethane was used to extract oils from water.

Method for rheological characterization. Rheological characterization was carried out on a modular compact rheometer (MCR302, Anton Paar) with a parallel plate (PP 15 mm) used to conduct oscillatory tests. The gel samples were cut into round plate with a diameter of 15 mm and a thickness of ~4 mm. Temperature-ramp experiments were carried out by cooling the samples at a rate of 3 °C /min from 20 °C to -25 °C, and then keeping at 20 °C for 20 mins at least, then heating samples at the same rate from 20 °C to 80 °C, while monitoring the viscoelastic moduli of both the steps under small-amplitude

oscillatory shear at an applied frequency of ω = 10 rad/s and strain amplitude of γ_0 = 0.1%. Frequency sweeps at 25 °C temperatures were carried out over a range of ω 0.1-20 rad/s at strain amplitude of γ_0 = 0.1%.

Compression test. The compressive stress-strain measurement and loading-unloading cycles were performed using universal tensile machine (Instron). The cylindrical gel samples with ~10 mm diameter and ~5mm thickness were set on the lower plate and compressed by the upper plate, at the rate of 5 mm per minute for compressive stress-strain measurement and 2 mm per minute for loading-unloading cycles.

Method for differential scanning calorimeter (DSC) characterization of dry polymer networks. A differential scanning calorimeter (TA-Q2000) was used to measure the thermal properties of the dry polymer networks. The DSC thermograms covered the temperature range of -30 to 200 °C at a scanning of 10 °C/min. All samples were pretreated under 80 °C overnight to dry state and the second scan curves were chosen to display, aiming to remove the negative effect of thermal history.

XRD measurements. The XRD measurements were performed on Xeuss WAXS/SAXS system, France. The sample to detector distance was 127 mm. The samples were measured at -15 °C degree using a Linkam LTS420 hot stage. The hydrogel sample was fabricated based on thermal polymerization H6 in Supplementary Table 1 which corresponded with the HYDROGEL sample for rheological characterization (weight ratio of water to HPN ~10.1). The organohydrogel sample was prepared based on H6 in Supplementary Table 1 and O2 in Supplementary Table 2 with weight ratio of water to HPN ~9.7 and n-decane to OPN ~0.2.

Polarized light microscopy. Light microscopy of ice crystals on hydrogel and organohydrogel was carried out using a Polarized Light Microscope (Nikon, Eclipse, Lv100N, POL) equipped with a polarizer and an analyzer.

Supplementary Equation 1

Swelling ratio Q:

$$Q = V/V_0 \times 100\%,$$

Where V is the volume of the samples in the equilibrium swollen state and V_0 is the initial volume of dry polymer networks.

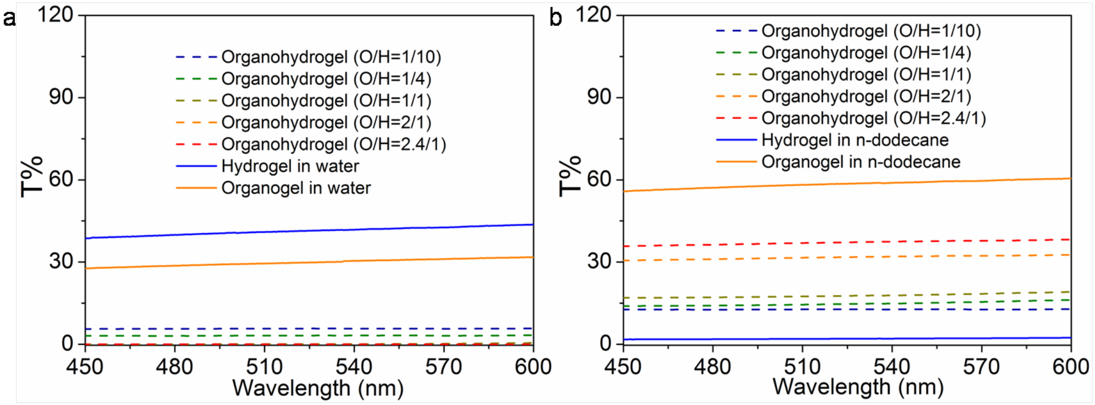

Supplementary Figure 1. The curves for transparency of organohydrogel in water **(a)** and in n-dodecane **(b)** with different OPN/HPN ratios *vs.* wavelength (450-600 nm) are shown.

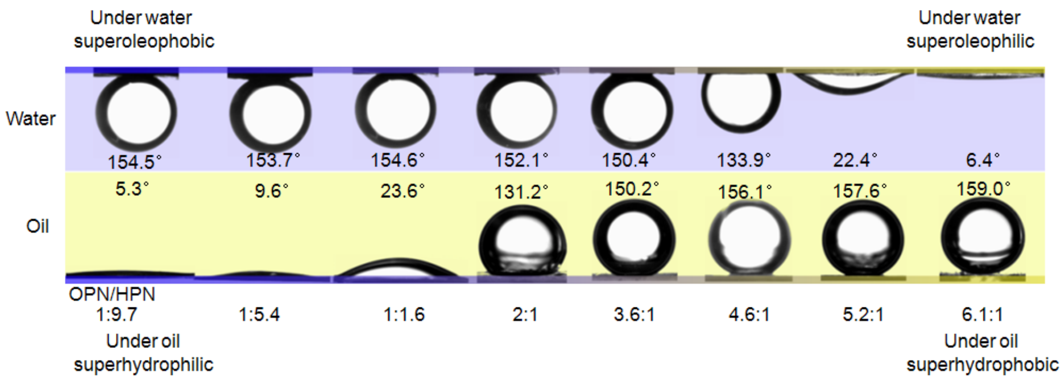

Supplementary Figure 2. Owing to the opposite solvent affinities of HPN and OPN networks, organohydrogel undergoes conformational rearrangements in selective solvents in a controlled fashion. Surface properties of organohydrogels can be controlled from superoleophobic to superoleophilic in water and from superhydrophilic to superhydrophobic in oil by tuning OPN/HPN ratio. The organohydrogel samples were prepared based on H3 in Supplementary Table 1and O1 in Supplementary Table 2.

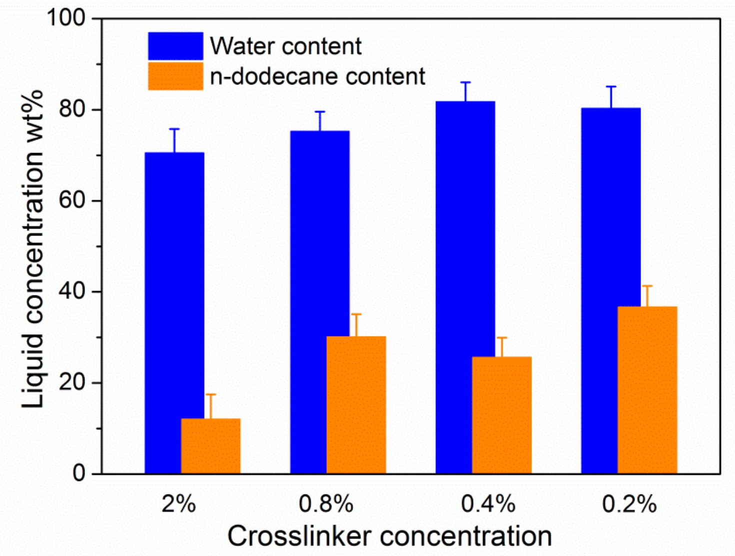

Supplementary Figure 3. The maximum water and oil contents in organohydrogel made up of hydrophilic networks with different crosslinker concentration. The water content is around 65-80 wt%, and the oil content is approximately 10-40 wt%. The error bar of the average separation efficiency was calculated from 3 parallel experiments.

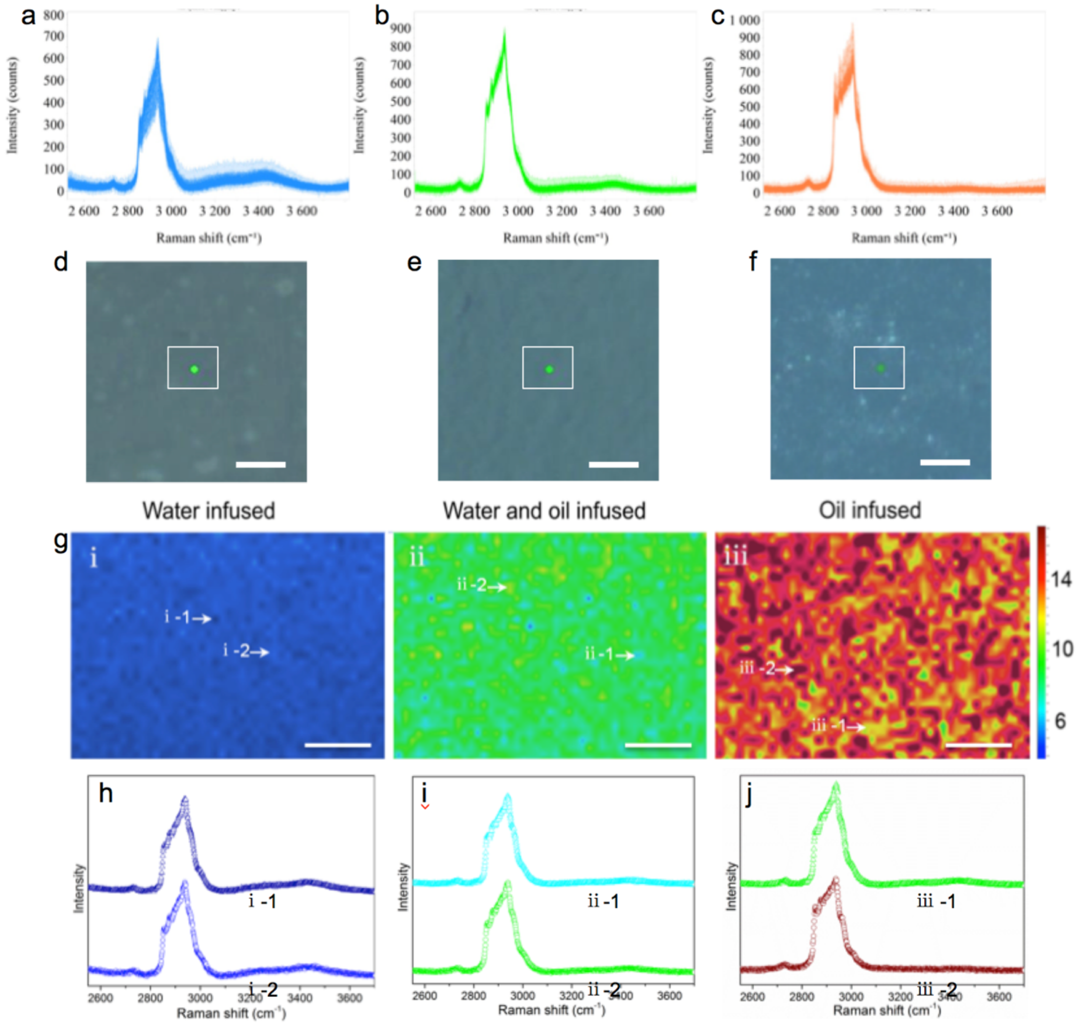

Supplementary Figure 4. Raman information of individual organohydrogel equilibrate with pure water (the left column), water and oil (the middle column), and pure oil (the right column). **a-c**, Raman spectra of all scanning points (≈ 2,000 points) in scanning range (8 μm×10 μm). **d-f,** The optical images of the scanning position. In micro-scale, all of the three surfaces are basically flat. The scale bar is 10 μm. **g,** The points with different color are marked in the three images **(i-iii).** The scale bar is 2 μm.; and the corresponding spectra are shown in **(h-j).**

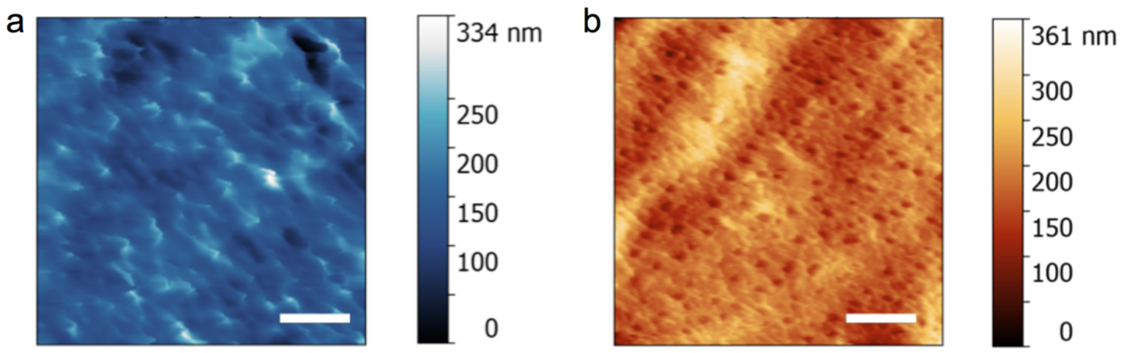

Supplementary Figure 5. AFM analysis of the organohydrogel surfaces were performed in different submerged environments to reveal the surface topography changes induced by the hetero-network reconfiguration. When the organohydrogel was immersed in water, rough structures with several hundred nm-sized domains formed on the surface, which was mainly attributed to the swollen HPNs **(a)**. In contrast, when immersed in n-dodecane, the hydrogel domains collapsed, forming pit structures with diameters around 200 nm on the surface **(b)**. Under these conditions, the exposed surface was covered by almost all organogel domains. The AFM scale bar is 1 μm.

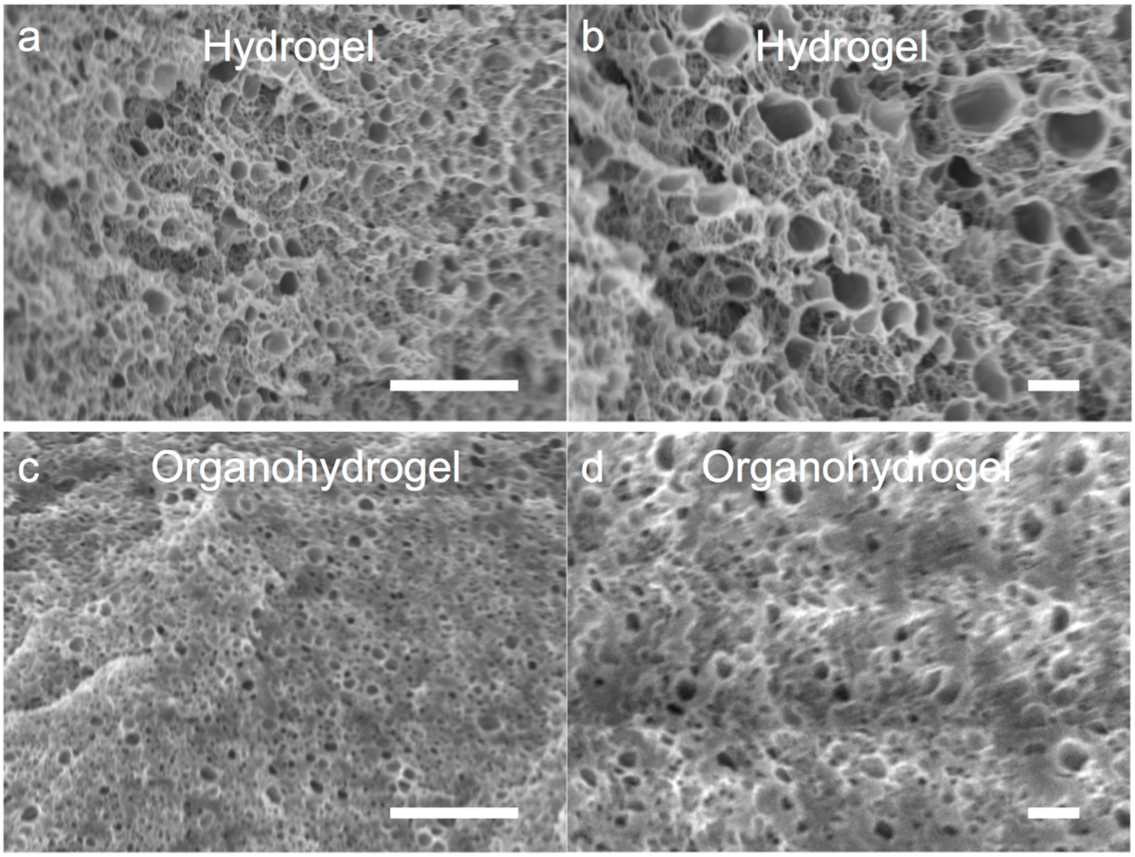

Supplementary Figure 6. The scanning electron microscopy images show the morphologies and porosity of hydrogel **(a-b)** and organohydrogel **(c-d)**. Both of them have evident porous structures. The pores on hydrogel are most at the micrometer scale. In contrast, fewer and smaller pores are observed on organohydrogel. The pores on organohydrogel are more uniform and most at several hundred nanometer scale, because of the filling effect of oleophilic networks. In Supplementary Figure6a and c, the scale bar is 5 μm. In Supplementary Figure6b and d, the scale bar is 1 μm.

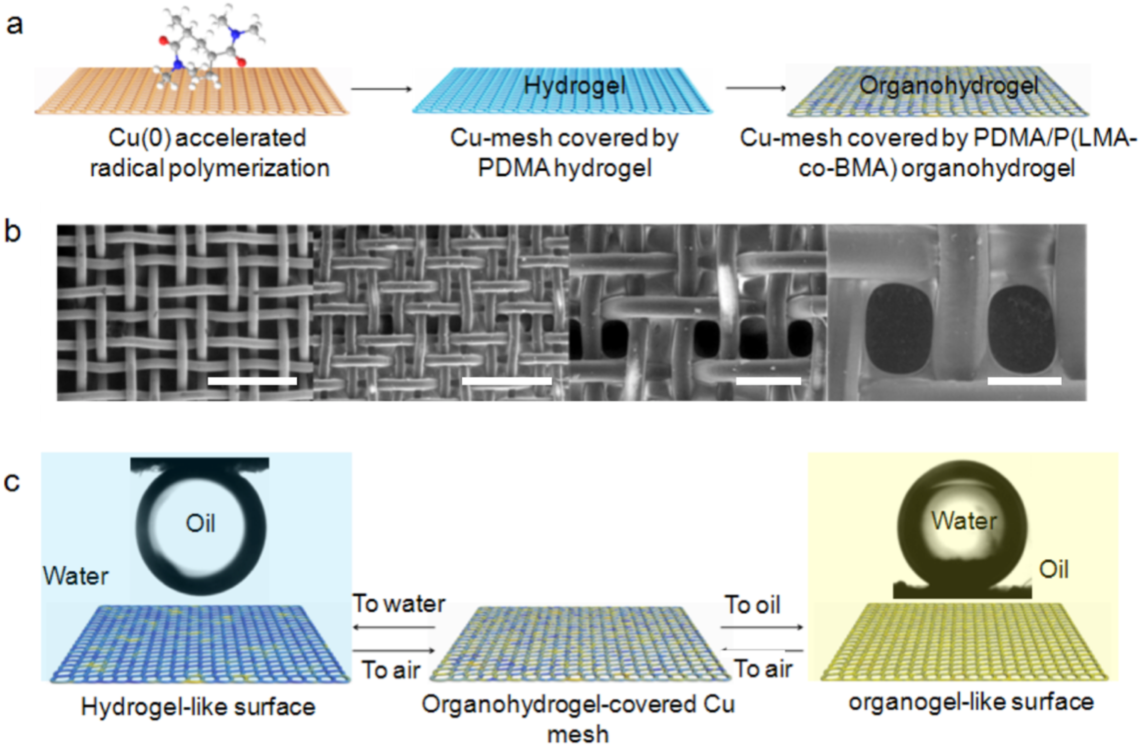

Supplementary Figure 7. a, The schematic of the preparation of organohydrogel-covered Cu mesh (the OPN/HPN ratio of organohydrogel is 2.0/1.0). **b,** The SEM image of organohydrogel-covered Cu mesh. The pore diameter is around 100 μm. The scale bar is 500 μm, 200 μm and 100 μm from left to right. **c,** The contact angle of oil droplet under water (152.5 ± 1.3°), and oil droplet under water (156.1 ± 1.9°) on mesh surface.

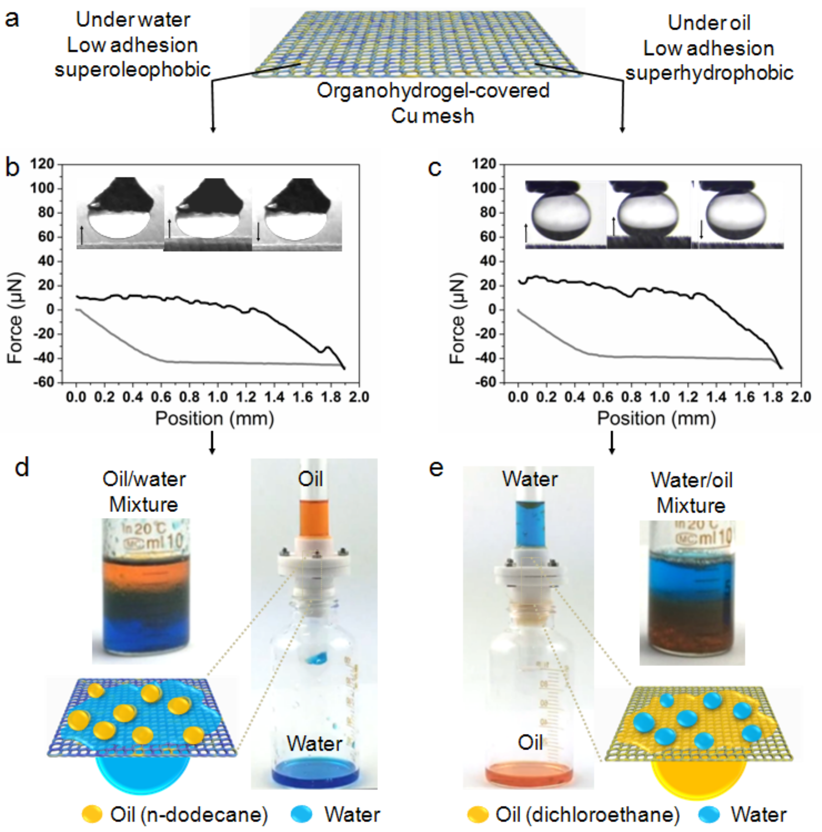

Supplementary Figure 8. The application of the self-adaptive organohydrogel mesh in smart liquid separation. We present proof-of-principle demonstrations utilizing the organohydrogel modified copper meshes (200 meshes) to carry out on-demand oil/water separation. **a,** the organohydrogel-covered Cu mesh was fabricated, which showed excellent superoleophobic/low adhesion to oil under water and superhydrophobic/low adhesion to water under oil. **b-c,** Photographs of the dynamic under-water oil adhesion and under-oil water adhesion measurements on the coated mesh and the corresponding measurements curves, showing the self-adaptive low adhesion characteristics. **d-e,** Photographs showing the self-adaptive separation of oil/water. The organohydrogel covered mesh was fixed between two glass tubes. Emulsible mixtures of n-dodecane and water (1:1 vol/vol) were poured onto an organohydrogel-coated cooper mesh that had been pre-equilibrated in water. The n-dodecane was blocked, while water penetrated the mesh and flowed through. Conversely, when pre-equilibrated in 1,2-dichloroethane, the organohydrogel-coated mesh successfully blocked water and allowed 1,2-dichloroethane to pass though easily.

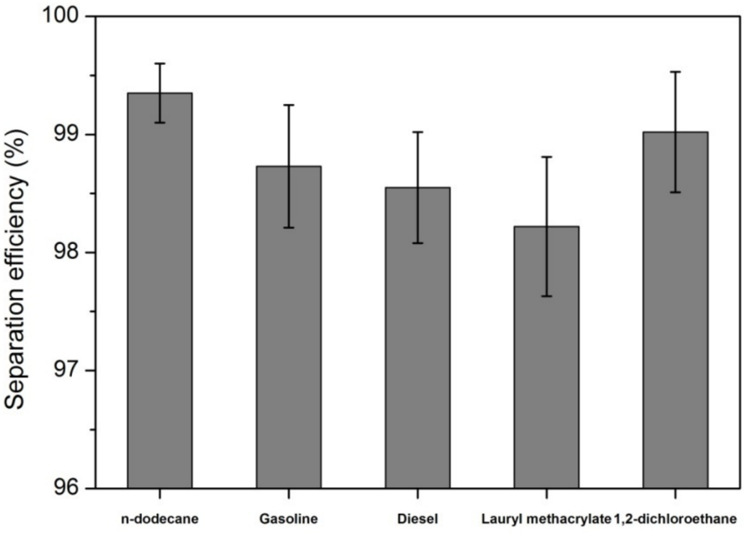

Supplementary Figure 9. The average separation efficiency of the organohydrogel-covered mesh is shown above. The mixture of water and n-dodecane, gasoline, diesel, and lauryl methacrylate were separated based on the mesh with hydrogel-like surfaces; while the mixture of water and 1,2-dichloroethane was separated based on the mesh with organogel-like surfaces. The error bar of the average separation efficiency was calculated from 3 parallel experiments.

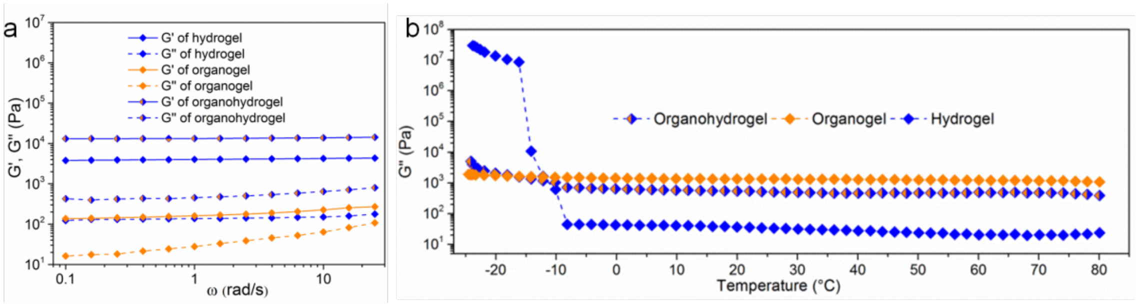

Supplementary Figure 10. a, Linear viscoelastic spectra (G’ and G’’) of the samples (hydrogel, orgohydrogel and organogel) at 25 °C. For organohydrogel with hetero-network, the G’ at room temperature is higher than hydrogel or organogel with homogeneous polymer networks, indicating the enhanced effect of interpenetrating OPN/HPN structures. The three gels contained almost same concentrations of polymer networks. Hydrogel 71 wt% water; organohydrogel 69 wt% liquid mixture of water (54 wt%) and n-decane (15 wt%); organogel 67 wt% n-decane. **b,** the G’’ of the samples (hydrogel, organohydrogel and organogel) from -20 to 80 °C, which is corresponding with G’ in Fig. 4a of the text.

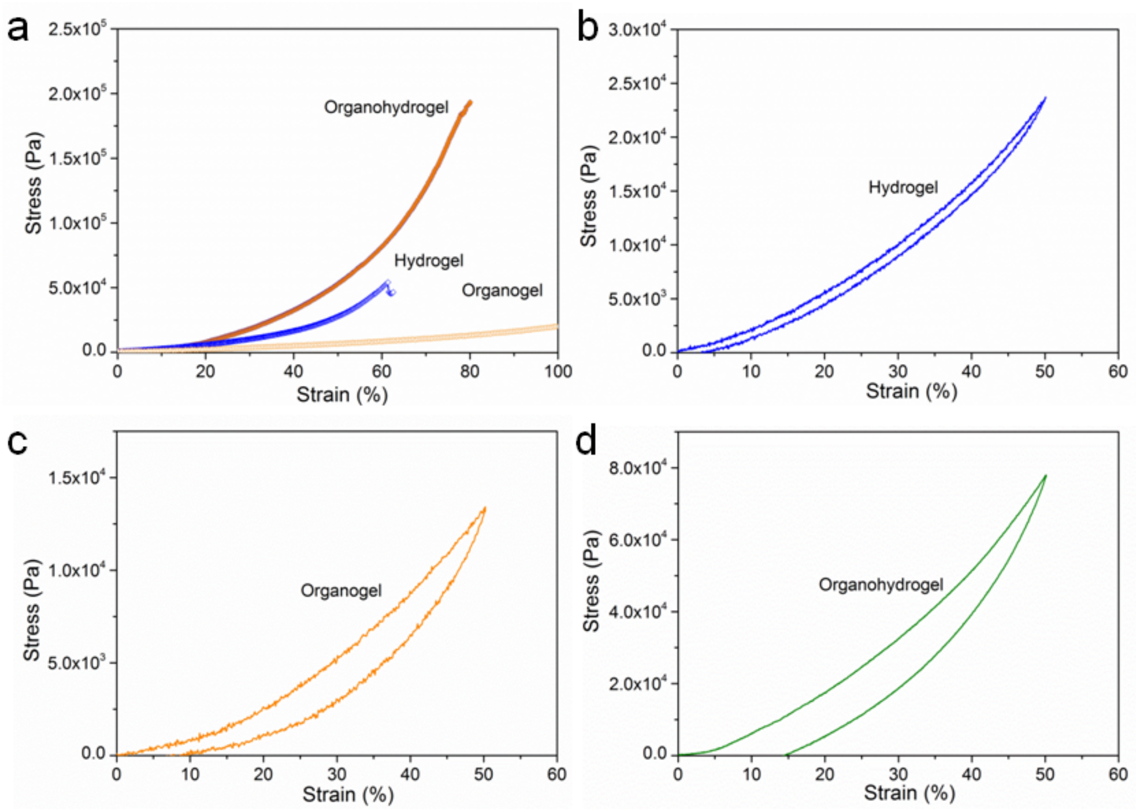

Supplementary Figure 11. a, Stress-strain curves for organohydrogel (58 wt% water and 8 wt% n-decane), hydrogel (93 wt% water), and organogel (15 wt% n-decane) under uniaxial compression. The organohydrogel can sustain up to 190 kPa compression; while, the hydrogel breaks at a stress of 54 kPa. **b-d,** the loading and unloading curves for the first cycle in compression, based on the samples of organohydrogel (58 wt% water and 8 wt% n-decane), hydrogel (93 wt% water), and organogel (15 wt% n-decane).

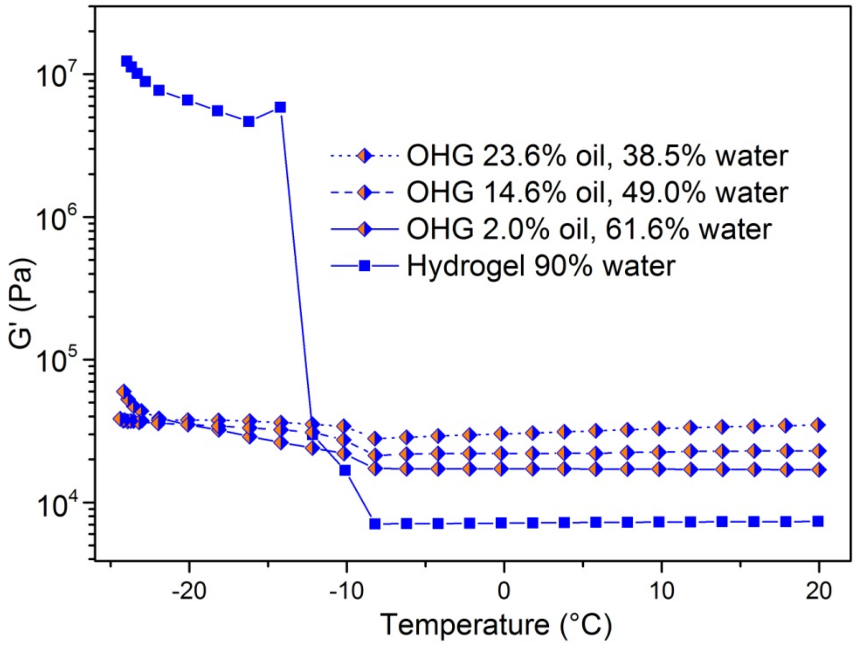

Supplementary Figure 12. G’ of organohydrogel (OHG) with different solvent contents and hydrogel with 90 wt% water as control. With the increase of the weight percent of oil (n-decane), the viscoelasticity of organohydrogels in subzero temperature would become more stable. When the organohydrogel containing 38.5 wt% water, the G’ only increased by 3.9% at -15 °C compared with that at 20 °C. Apparently, the increase of G’ at subzero temperature was due to the freezing of hydrophilic networks. However, the oleophilic networks were still able to maintain the elasticity at subzero temperature. More oil infused caused the elasticity of the organohydrogels under subzero temperature to become more stable.

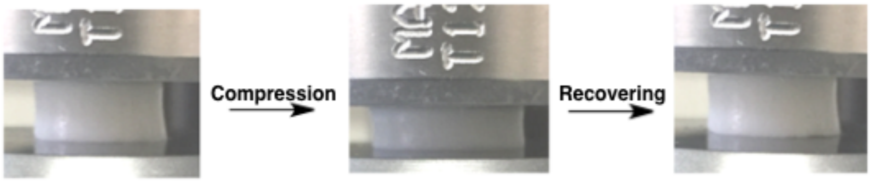

Supplementary Figure 13. The loading and unloading curves for the first cycle in compression, based on the samples of organohydrogel (~18% n-heptane and ~10% water).

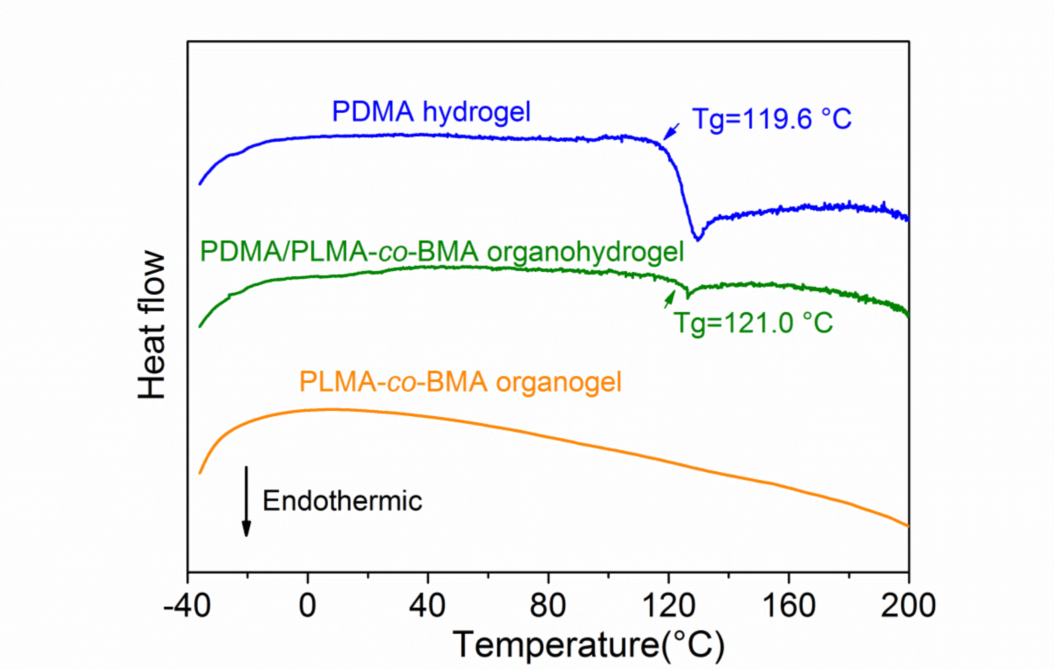

Supplementary Figure 14. DSC curves measured at a heating rate of 10 °C/min from -30 °C to 200 °C for dry polymer networks of PDMA, PLMA-co-BMA, and hetero-networks of organohydrogel. The glass transition temperature (Tg) of PDMA (based on H6 in Supplementary Table 1) is 119.6 °C. When the temperature rises up to ~40 °C, the evident and long-range endothermic phenomena of PLMA-co-BMA are observed; because the polymer networks of PBMA chain segments (Tg ~40 °C) start its softening[3]. However, the hetero-networks of organohydrogel show relatively stable curve without apparent decalescence below 80 °C. The endothermic peak of hetero-networks of organohydrogel at 121 °C corresponds to the Tg of PDMA networks.

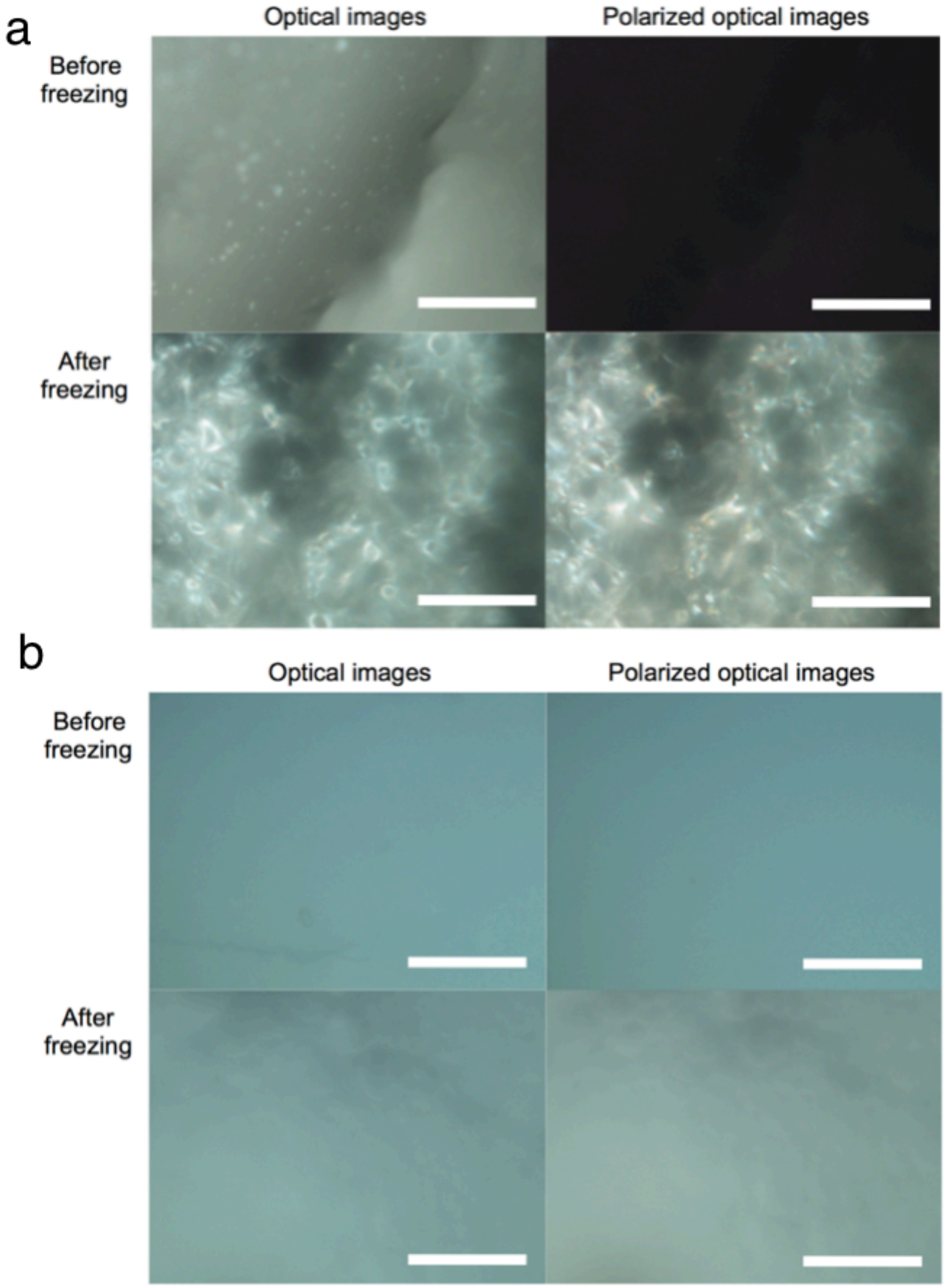

Supplementary Figure 15. The photographs based on polarization microscopy show that **a,** the surface of PDMA hydrogel before and after freezing. We can clearly see that the ice crystals forming on the surface after freezing. **b,** the surface of organohydrogel before and after freezing. The organogel components poly(BMA-co-LMA) produce polarization phenomenon intrinsically thus the polarized light of organohydrogel before freezing show

a certain brightness. By contrast with hydrogel, the images of organohydrogel after freezing do not reveal remark bulk ice crystals. The scale bar is 50 μm.

Supplementary Table 1. Polymerization of PDMA hydrogel

No.	DMA (g)	BIS (g)	APS (g)	DI-Water (g)
**H1**	5	0.2	0.05	20
**H2**	5	0.1	0.05	20
**H3**	5	0.05	0.05	20
**H4**	5	0.033	0.05	20
**H5**	5	0.025	0.05	20
**H6**	5	0.05	0.05	28.3

N,N-dimethylacrylamide (DMA), Methylene-bis-acrylamide (BIS), Ammonium persulphate (APS).

Supplementary Table 2. The precursors of organohydrogel based on poly(BMA-co-LMA) networks

No.	BMA (mL)	LMA (mL)	Ethanol (mL)	EGDMA (mL)	DEAP (g)
**O1**	10	10	10	0.15	0.1
**O2**	5	15	10	0.01	0.1

n-butyl methacrylate (BMA), Lauryl methacrylate (LMA), Ethylene glycol dimethacrylate (EGDMA), 2,2-diethoxyacetophenone (DEAP).
